# A trial assessing N-3 as treatment for injury-induced cachexia (ATLANTIC trial): does a moderate dose fish oil intervention improve outcomes in older adults recovering from hip fracture?

**DOI:** 10.1186/1471-2318-10-76

**Published:** 2010-10-22

**Authors:** Michelle D Miller, Alison Yaxley, Anthony Villani, Lynne Cobiac, Robert Fraser, Leslie Cleland, Michael James, Maria Crotty

**Affiliations:** 1 Nutrition and Dietetics, Flinders University, GPO Box 2100, Adelaide SA 5001, Australia; 2 Rehabilitation and Aged Care, Flinders University, GPO Box 2100, Adelaide SA 5001, Australia; 3 Department of Medicine, Flinders University, GPO Box 2100, Adelaide SA 5001, Australia; 4 Rheumatology Unit, Royal Adelaide Hospital, North Terrace, Adelaide SA 5000, Australia

## Abstract

**Background:**

Proximal femoral fractures are associated with increased morbidity and mortality. Pre-existing malnutrition and weight loss amongst this patient group is of primary concern, with conventional nutrition support being largely ineffective. The inflammatory response post proximal femoral fracture surgery and the subsequent risk of cachexia may explain the inability of conventional high energy high protein management to produce an anabolic response amongst these patients. Omega-3 fatty acids derived from fish oils have been extensively studied for their anti-inflammatory benefits. Due to their anti-inflammatory properties, the benefit of fish oil combined with individualized nutrition support amongst proximal femoral fracture patients post surgery is an attractive potential therapeutic strategy. The aim of the ATLANTIC trial is to assess the potential benefits of an anti-inflammatory dose of fish oil within the context of a 12 week individualised nutrition program, commencing seven days post proximal femoral fracture surgery.

**Methods/Design:**

This randomized controlled, double blinded trial, will recruit 150 community dwelling elderly patients aged ≥65 years, within seven days of surgery for proximal femoral fracture. Participants will be randomly allocated to receive either a 12 week individualized nutrition support program complemented with 20 ml/day anti-inflammatory dose fish oil (~3.6 g eicosapentaenoic acid, ~2.4 g docosahexanoic acid; intervention), or, a 12 week individualized nutrition support program complemented with 20 ml/day low dose fish oil (~0.36 g eicosapentaenoic acid, ~0.24 g docosahexanoic acid; control).

**Discussion:**

The ATLANTIC trial is the first of its kind to provide fish oil combined with individualized nutrition therapy as an intervention to address the inflammatory response experienced post proximal femoral fracture surgery amongst elderly patients. The final outcomes of this trial will assist clinicians in the development of effective and alternative treatment methods post proximal femoral fracture surgery which may ultimately result in a reduction in systemic inflammation, loss of weight and lean muscle and improvements in nutritional status, mobility, independence and quality of life among elderly patients.

**Trial Registration:**

ACTRN12609000241235

## Background

Osteoporotic fractures and injurious accidental falls are associated with increased morbidity and mortality in older adults [1-4]. Proximal femoral fractures (PFF) are among the most common causes for acute admission to orthopaedic wards with a 30% mortality rate 12-months post surgery [3]. Moreover, less than 50% of patients return to pre-fracture levels of mobility and independence, with older adults who survive a hip fracture being three times more likely to be functionally dependent, 50% requiring long-term assistance with routine functional activities and up to 25% requiring full-time residential care [5-7].

Malnutrition in the elderly is commonly overlooked in a clinical setting [8,9]. There is an extremely high prevalence (up to 63%) of pre-existing malnutrition amongst PFF patients on acute admission to hospital [9,10]. Malnutrition in this setting has consistently been demonstrated to lead to adverse patient outcomes through increasing the risk of postoperative complications [11], limiting participation in rehabilitation and delaying optimal recovery of ambulatory status and independence [12,13]. Moreover, there is a marked decline in nutritional status in these patients throughout the acute admission, commencing within one week of admission and continuing for six to twelve weeks post surgery [14,15].

Conventional nutritional management of PFF patients involves the provision of high energy, high protein diets, however the evidence for this approach is largely unconvincing. A systematic review of the literature highlighted that most nutrition support provided for PFF patients is based on the provision of a standard volume of high caloric oral supplement rather than individualised dietary requirements [16]. One of the primary limitations of a standard approach is the failure to be patient orientated and tailor dietary therapy to each individual's energy and protein deficits associated with pre-existing malnutrition and inadequate dietary intakes post PFF surgery [16].

Weight loss in older adults, particularly loss of lean muscle, is highly predictive of increased morbidity and mortality [17-19]. Furthermore, rapid deconditioning of muscle may be responsible for secondary hospital readmissions, repeat injurious falls and loss of independence in older adults [20,21]. Cachexia, characterized by disproportionate muscle wasting, associated with systemic inflammation and elevated plasma cytokines [17], is a plausible explanation for the inability of conventional nutrition support programs to generate an anabolic response in PFF patients. It is critical, therefore, that caloric and protein supplementation is supported with other aggressive treatment strategies in the treatment of cachexia [22,23].

As a biomarker of cachexia, the magnitude of the inflammatory response post PFF surgery, and interventions to reduce this response, has not been adequately investigated. The normal physiological response to a major insult, such as the trauma of PFF and the subsequent surgical repair, is characterized by the activation of the hypothalamic pituitary adrenal axis and sympathetic nervous system with simultaneous activation of the immune system and systemic inflammation [24]. Persistent inflammation among patients recovering from PFF is recognized as a major contributing factor to poor patient outcomes post surgery [25]. Specifically, the combination of stress hormones and pro-inflammatory cytokines promotes catabolism and malnutrition, and impairs normal gastrointestinal functioning such that macronutrient utilization may be compromised [26]. Moreover, inflammation in this population group is predictive of a decrease in physical functioning, the onset of disability and mortality [27-30].

Due to their anti-inflammatory properties and potential to suppress the metabolic consequences of PFF, fish oils are a relatively simple and promising therapeutic strategy for the treatment of cachexia in this population group. Provision of adequate fish oil in conjunction with adequate energy and protein could theoretically improve outcomes for PFF patients. Fish oils, more specifically eicosapentaenoic (EPA) and docosahexanoic (DHA) acids, have been extensively studied for their anti-inflammatory properties in inflammatory conditions including rheumatoid arthritis [31] and cardiovascular disease [32]. However, the novel therapeutic benefit of fish oils in patients with end-stage disease is also attracting wider attention with research suggesting that fish oils may ameliorate cachexia in patients with advanced cancer [33]. One proposed mechanism of the anti-inflammatory action of fish oils is impairment of the release of acute phase proteins and pro-inflammatory cytokines, in particular C-reactive protein (CRP), interleukin-6 (IL-6), tumor necrosis factor-∝ (TNF-∝) and interleukin-1 (IL-1) [34].

The aim of the present randomised controlled trial (RCT) is to implement a 12 week, individualised nutrition program complemented with an anti-inflammatory dose of fish oil (20 ml/day containing 3.6 g EPA and 2.4 g DHA), commencing 7 days post PFF surgery that will result in clinically important and statistically significant improvements in terms of quality of life, appetite, body composition and physical function. Comparisons will be drawn with a control group receiving 10% of the active fish oil dose.

## Methods/Design

### Design overview

**Figure 1:**
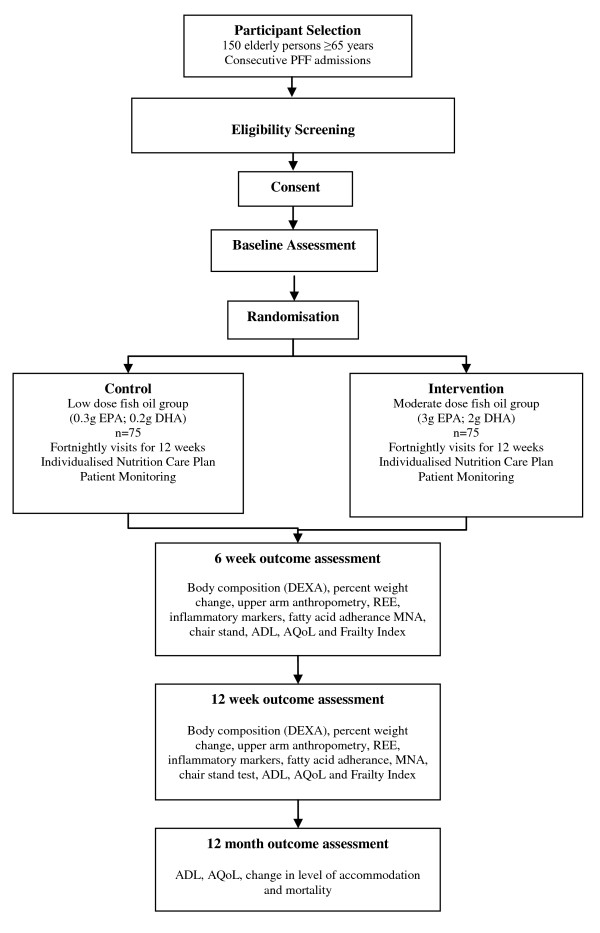
**Study design and assessment points**. Abbreviations: PFF - proximal femoral fracture; EPA - eicosapentaenoic acid; DHA - docosahexanoic acid; DEXA - dual energy x-ray absorpitometry; REE - resting energy expenditure; MNA - mini nutritional assessment; ADL - activities of daily living; AQoL - assessment of quality of life instrument.

This is a double blinded randomized controlled trial of 150 adults aged ≥65 years, recruited within seven days of surgery for PFF and followed post discharge for up to 12 months. Participants identified with cachexia will be randomly allocated to receive either a 12 week individualized nutrition program complemented with an anti-inflammatory dose of fish oil (20 ml/day providing ~3.6 g EPA and ~2.4 g DHA per day) (**intervention)**, or, a 12 week individualized nutrition program complemented with 20 ml/day low dose fish oil (~0.36 g EPA, ~0.24 g DHA) (**control**). An overview of the recruitment method, randomisation process and follow-up procedures is provided in Figure 1. This trial has been approved by the Human Research and Ethics Committee (HREC) at the Royal Adelaide, Flinders Medical Centre and Flinders Private Hospitals, and registered with the Australian and New Zealand Clinical Trials Registry (reg no: ACTRN12609000241235).

### Recruitment and Eligibility

Participants will be consecutively recruited from admissions to the acute orthopaedic wards at Flinders Medical Centre, Flinders Private Hospital and the Royal Adelaide Hospital, Adelaide, South Australia. Project personnel, in consultation with members of the orthogeriatric team, will screen all orthopaedic patients against eligibility criteria, outlined in Table 1. All participants will be recruited within 7 days of PFF surgery, with the intervention to commence on day 7 post surgery for repair of PFF.

**Table 1:** Eligibility criteria for ATLANTIC trial

Inclusion criteria	Exclusion criteria
Aged ≥65 years	Severe cognitive impairment (MMSE <18/30)
PFF confirmed by radiology report	PFF not fall-related, is pathological or is peri-prosthetic
Undergone surgery for fixation of hip fracture	Initiation of anti-inflammatory drugs or corticosteroids within the previous two weeks
Reside within existing local service boundaries	Ingestion of ≥2 g total omega-3 daily as per fish FFQ
Preliminary evidence of cachexia (CRP ≥6 mg/L)	Seafood allergy
At risk of significant weight loss (SNAQ score <14/20)	Life expectancy <12 weeks
	Inability to communicate effectively due to ■ Blindness ■ Deafness ■ Lack of a translator
	Inability to provide informed consent
	Diagnosed with one of the following haemorrhagic stroke risk factors Unstable hypertension ■ Atrial fibrillation ■ Inherited disorders - polycystic kidney disease, Ehlers-Danlos syndrome type IV, neurofibromatosis type I, Marfan syndrome, moyamoya disease
	Diagnosed with a bleeding disorder such as the following: ■ von Willebrand disease ■ Haemophilia
	Receiving treatment doses of any antithrombotic agent
	Those on dual anti-platelet function drugs or any anti-platelet drugs with exception of low dose aspirin.

Abbreviations: MMSE - Mini Mental State Examination; PFF - proximal femoral fracture; FFQ - food frequency questionnaire; CRP - C-reactive protein.

Medical records will be audited to confirm age, suburb of residence and details of injury. A complete medication history, relevant biochemical analyses, information on risk factors for haemorrhagic stroke or increased bleeding times, and co-morbidities likely to reduce life expectancy to <12 weeks will be collected from each participant prior to recruitment. With exception to low-dose aspirin and prophylactic doses of low molecular weight (LMW) heparin, patients receiving treatment doses of any antithrombotic agent, including those taking anti-platelet functioning drugs, will not be eligible to participate in the study. To identify preliminary evidence of cachexia, patient serum CRP levels >6 mg/L will be used as the standard in screening prospective participants.

A validated fish food frequency questionnaire (FFQ) [35] will be administered for the purposes of estimating current intake of n-3 fatty acids. Participants with a daily intake of >2 g total n-3 fatty acids as per FFQ will be deemed ineligible to participate in the present study. Any seafood allergy documented in patient medical records will be noted but confirmed with the patient and family if the patient is otherwise eligible.

Risk of subsequent weight loss will be assessed in the recruitment of participants using the validated Simplified Nutrition Appetite Questionnaire (SNAQ), a four item questionnaire derived from the eight item Council on Nutrition Appetite Questionnaire [36]. SNAQ investigates appetite, satiety following meals, food taste and usual meal frequency. Responses are scored on a five-point scale with a score <14/20 representing a significant risk of weight loss. Participants who are deemed not at risk of significant weight loss (i.e. score ≥14/20 weight) will be ineligible for the present study.

The Mini Mental State Examination (MMSE) [37] will be administered to assess cognitive impairment and those who achieve <18/30 will be excluded due to their inability to independently follow therapeutic advice and difficulty in verification of self-reported outcomes. When assessing MMSE, any patient who does not score ≥18/30 on the first attempt post surgery, but is suspected of experiencing post-operative delirium, will be reassessed at a later date (within 7 days of surgery) in order to maximise the opportunity of achieving a score ≥18/30. For prospective participants who score between 18 and 24/30, consent will also be obtained from next of kin to confirm participation.

All patients who meet the eligibility criteria will be invited to participate in this trial. Project personnel will provide potential participants with a detailed description of the study and the procedures to be performed. If research personnel are satisfied that the potential participant completely comprehends the information and the implications of their involvement in the study, written informed consent will be obtained prior to commencement of baseline measures and randomisation. If study personnel have any doubts that a participant has the capacity to give informed consent, third party consent will be obtained from a relative or immediate caregiver.

### Measurements and Procedures

Project personnel will assess the selected outcome measures (outlined below) at baseline, 6 weeks and 12 weeks with selected measures at 12 months. Project personnel will remain masked to treatment allocation for the assessment of all outcome measures. A telephone follow-up will be performed at 12-months to collect information on quality of life, physical autonomy, changes in level of accommodation and mortality. Outcome measures have been selected based on their suitability for older adults following PFF surgery, strength of the evidence to support validity and reliability, and significance to rehabilitation. All outcome assessments will be conducted in a clinic setting. For all participants who have been discharged post PFF surgery, transportation will be provided by project personnel. It is anticipated that outcome assessments will take approximately 90 minutes in duration.

#### Body composition and nutritional status

##### Lean body mass and bone mineral density (baseline, 6 and 12 weeks)

Lean body mass and bone mineral density will be measured using Dual Energy X-ray Absorptiometry (DEXA: Lunar Prodigy Pro, GE Healthcare, UK). Loss of lean body mass is a key feature in the development of cachexia therefore assessment of the effect of fish oil on body composition is important. Bone mineral density (Anterio-Posterior (AP) spine and hip) will also be examined.

##### Percent weight change (baseline, 6 and 12 weeks)

Weight change is a key feature in the monitoring of recovery from cachexia. Body weight will be measured to the nearest 0.1 kg following an overnight fast, using calibrated electronic digital scales (Tanita, BF-679W, Illinois, United States) with patients in light clothing and without footwear. Alternatively, for inpatients at baseline, a calibrated weigh chair will be used. Percent weight change will be determined by calculating the difference from baseline weight, divided by baseline weight, multiplied by 100. Research Dietitians visiting participants fortnightly will also weigh participants routinely as a means of monitoring patient progress and nutritional status.

##### Nutritional status (baseline, 6 and 12 weeks)

The Mini Nutritional Assessment (MNA), a validated nutrition screening and assessment tool, will be used to classify patients as well-nourished, at risk of malnutrition or malnourished [38]. This assessment collects information from four main domains: anthropometric measures (body mass index (BMI) and self reported weight loss), global assessment (lifestyle, medications and mobility), dietary assessment (energy and protein intake and number of meals), and subjective assessment (self-rated health and nutritional status).

Triceps skinfold thickness will be assessed using calibrated skinfold calipers (Harpenden calipers, Baty International, Sussex, United Kingdom) and combined with mid-arm circumference to determine corrected arm muscle area (CAMA) and thereby estimate skeletal muscle mass [37].

##### Resting energy expenditure (baseline, 6 and 12 weeks)

Resting energy expenditure (REE) will be measured using a hand held portable indirect calorimeter (MedGem, Health-Tech, Germany). REE will be assessed following a fasting period of at least 4 hours. While sitting in a relaxed and upright position, participants will be required to breathe normally into a disposable "scuba-type" mouthpiece where oxygen consumption will be measured until steady state breathing is detected.

#### Physical function

##### Physical autonomy (baseline, 6 and 12 weeks)

The ability to participate in activities of daily living (ADL) will be assessed using a validated instrumental and physical ADL tool [39]. The instrumental ADL assesses the ability to shop, prepare meals, conduct housework duties, wash clothing, use the telephone, manage finances and take medications, whereas the physical ADL tool assesses the ability to eat, dress, shower, get into and out of bed, maintain self-appearance and walk. Physical autonomy is considered an important requirement of independent living, where poor performance is an established risk factor for morbidity and mortality amongst older adults [39,40].

##### Chair stand test (baseline and 12 weeks)

This assessment correlates with lower limb muscle strength, which is an integral component of physical independence amongst the elderly population [41]. Participants will be assessed using a validated physical performance test [42] where they will be instructed to perform a timed test of five repetitions of rising from a seated position and subsequently returning to their original seated position as quickly as possible. The standard protocol has been amended slightly to account for suitability within this patient group. Specifically, participants will be asked to perform the test on two separate occasions, once with the use of arms (i.e. using arm rests to push off chair) and secondly, as per protocol, without the use of arms (i.e. with arms folded across chest).

#### Laboratory assessment

##### Biochemical markers of inflammation (baseline, 6 and 12 weeks)

Fasting blood samples will be collected by a trained research nurse at each of the outcome assessments. Serum samples will be obtained, processed and stored frozen at -70°C. When stored at such temperatures, there is evidence to suggest inflammatory cytokines such as IL-6 remain stable in serum for many years [43]. The analyses of inflammatory markers including CRP, TNF-∝, IL-1β, IL-6 will be assayed using commercial cytokine enzyme-linked immunosorbent high-sensitivity kits.

##### Adherence to the fish oil intervention (baseline, 6 and 12 weeks)

Whole blood samples will be collected and processed allowing for the separation of plasma from red blood cells. Fatty acids will subsequently be extracted from the erythrocyte phospholipid membrane and analysed to evaluate adherence to the fish oil intervention.

Additionally, study participants will be required to maintain a diary outlining their consumption of fish oil, which will then be monitored by the research Dietitian at fortnightly visits; any remaining fish oil will be measured to monitor participant adherence.

#### Other assessments

##### Quality of life (baseline, 6 and 12 weeks; 12 month follow-up)

Health-related quality of life will be assessed using the Assessment of Quality of Life Instrument (AQoL) [44], a 15 item survey with high internal consistency covering five domains: illness, independent living, social relationships, physical senses and psychological well-being. The resultant summary score can be transformed into a utility index, and subsequently, quality-adjusted life years calculated.

##### Frailty index (baseline, 6 and 12 weeks)

Frailty will be assessed using the Edmonton Frailty Index [45] a valid and reliable survey that can be used routinely by non-geriatricians, covering nine domains: cognition, general health status, social support, functional independence, nutrition, medication use, mood, continence, and functional performance.

#### Monitoring adverse effects to fish oil

##### Gastrointestinal side effects

Any gastrointestinal side effects of the fish-oil, although unlikely at the proposed dose, will be self-reported by participants at fortnightly visits, and include the monitoring of gastrointestinal symptoms such as nausea, vomiting, diarrhoea, abdominal pain and steatorrhoea. If participants are unable to tolerate the proposed dose, fish oil capsules will be offered at the equivalent dose. Alternatively the dose will be progressively reduced until any side effects have been resolved.

##### Interactions with medications

Prophylactic use of LMW heparin or low-dose aspirin will be carefully monitored by research personnel at baseline and throughout fortnightly visits. If any adverse events are observed, such as frequent bruising or increased bleeding time, these will be documented and reviewed by the trial safety management board and further reported to the human research ethics committee. If prophylactic doses for any anticoagulant medication increases to a treatment dose, the administration of fish oil will cease immediately. However, in a previous clinical trial of cardiac patients who were taking an anti-inflammatory dose of fish oil, in addition to aspirin and Warfarin therapy, no increase in bleeding episodes were observed [46]. Although the potential interaction between fish oil and antithrombotic agents among elderly cachectic patients has not been adequately examined, as a precautionary measure, patients taking treatment doses of anticoagulants are excluded from the present study

### Randomisation and Blinding

Participants will be randomly assigned to either an intervention or control group following the completion of all baseline measurements. Allocation to groups will be managed by the Flinders University statistician who provides a randomisation service using computerised randomisation software. Allocation will be stratified by hospital admission site due to the likely difference in services provided across the public and private settings. Allocation will also be stratified by fish intake (>2 oily fish meals/week; ≤ 2 oily fish meals/week) as determined by the validated fish FFQ [35]. This is a double-blinded controlled trial with all research personnel to remain masked to the treatment allocation of participants.

### Intervention

#### Individualised nutrition care plan

All participants, independent of group allocation, will receive a 12 week well-integrated, coordinated, individualised nutrition care plan in addition to a daily dose of either an anti-inflammatory dose or low dose of fish oil. The provision of the nutrition care plan is designed to ensure that both groups receive best available nutrition care for the prevention of starvation-related muscle wasting and hence will allow the study to evaluate whether an anti-inflammatory dose of fish oil improves outcomes beyond those achieved through ideal nutrition alone.

The research Dietitian will perform a comprehensive nutritional assessment upon each participant's entry into the study as a basis for developing a nutrition care plan consistent with estimated nutritional requirements and nutritional rehabilitation goals. As a basis for developing a nutrition program that will achieve positive energy balance, and therefore prevent clinically significant weight loss, REE of each participant will be measured. A factorial method, accounting for injury and weight gain factors, will be used to estimate the total energy expenditure for each participant. Nutrient Reference Values [47] will be used as a basis for determining macronutrient and micronutrient requirements.

To assess dietary intake, the research Dietitian will perform a standardised dietary interview (3-pass 24-hour recall) with each participant to determine total energy and nutrient intake. Strategies for achieving energy and nutrient requirements will include dietary counselling with attention to timing, size and frequency of meals, recommendations for nutrient dense foods, provision of recipes and referral to community meal programs and providers. Supplementation with commercial high-energy, high-protein supplements as well as multi-vitamins will also be considered to achieve optimal nutritional status.

#### Fish oil supplementation

For maximum adherence and minimal cost, administration of EPA and DHA will occur via unencapsulated fish oil taken on fruit juice or vegetable juice. Participants will be required to float 20 ml fish oil (Melrose Omega 18/12 - Aust L93186; Melrose Health, Victoria, Australia) on 30-40 ml juice, or any other flavoured, non-fizzy drink. The fish oil concentrate is flavoured with lemon and lime to mask the fish scent and taste. To avoid any potential side effects associated with the consumption of fish oil, participants are asked to consume the fish oil on juice with breakfast to avoid consumption on an empty stomach. If this dosage is not tolerated, the frequency and volume may be adjusted in conjunction with the research Dietitian, or alternatively fish oil capsules will be offered as an alternative to provide similar concentrations of EPA and DHA as the liquid oil (a total of 17 capsules per day, Melrose - Omega, Aust L156237; Melrose Health, Victoria, Australia).

The anti-inflammatory dose of fish oil selected for the intervention group provides ~3.6 g EPA and ~2.4 g DHA per 20 ml. The low-dose fish oil selected for the control group will be prepared to contain 10% of the active dose, which is also flavoured with lemon and lime to assist in maintaining participant blinding (~0.36 g EPA and ~0.24 g DHA per 20 ml). As with the intervention group, those participants in the control group who refuse to continue with the allocated dose of liquid oil will be offered fish oil capsules providing the equivalent dosage of EPA and DHA.

As an inpatient, at baseline, the participant's fish oil will be dispensed by each hospital's pharmacy and administered on the inpatient medication round. The pharmacy will also dispense outpatient supplies alongside other discharge medications.

#### Fortnightly visits

The research Dietitian will perform fortnightly home visits to all participants (intervention and control) over 12 weeks to perform dietetic care and maximise participant nutritional status by way of reviewing the nutrition care plan, dietary counselling and education, monitoring participant weight, ensuring energy and nutrient requirements are achieved as well as monitoring potential gastrointestinal side effects from the dose of fish oil.

### Sample Size Considerations

Quality of life is the primary outcome in this study and was selected because quality of life is an important determinant of future outcomes. It is recommended that half the baseline standard deviation of raw scores provides an appropriate estimate of the minimally clinically important difference for quality of life, and this form of estimation is useful in the absence of direct measurements of important change [48]. Using this method, the minimally clinically important difference was calculated to be approximately 2 points for the AQoL. A sample size of 64 participants per group is expected to achieve this difference between the two groups (power 80%, alpha = 0.05). To allow for deaths and withdrawals (5% and 10% respectively), 150 participants will be recruited (75 per group). This sample size will provide 92% power to detect a difference of 3 (effect size of 0.6) on the appetite questionnaire.

### Statistical Analysis

All statistical analyses will be performed using SPSS for Windows, version 18 (SPSS Inc, Chicago, USA). Data will be entered into a centralised secure database with remote web access (CareSearch Research Data Management System - http://www.caresearch.com.au) and will subsequently be exported into SPSS software for analyses. Primary analysis for this study will be undertaken using intention to treat principles. Independent samples t-tests, Mann-Whitney U test and Chi-square test of association will be used as appropriate to compare groups at baseline. To determine differences between the groups at the primary end-point (12 weeks), ANCOVA or logistic regression will be used with models adjusted according to potential confounders.

### Time Frame

This study has a 3.5-year time frame. It is expected that recruitment will commence late 2010 and continue until mid 2012. An additional 12 months will then be required to finalise data collection and six months to complete data analysis and prepare manuscripts for submission to peer reviewed scientific journals.

## Discussion

To the best of our knowledge, the ATLANTIC trial is the first of its kind to provide fish oil and individualised nutrition therapy as an intervention to address the deterioration in nutritional status which commonly occurs in PFF patients. This trial will explore more detailed information about the key markers of cachexia than previous work in this population group, particularly concerning the magnitude of inflammation experienced post PFF.

This trial will commence earlier than previous research where fish oil has been explored in the treatment of cancer cachexia. Given that the natural process of bone healing is influenced by a variety of systemic and local factors, including an early inflammatory phase [49], the intervention will commence day 7 following PFF surgery with an ongoing intervention phase of 12 weeks. The use of anti-inflammatory medication throughout the first week of bone healing may alter this natural inflammatory process and potentially inhibit normal bone healing [49]. Furthermore, the literature provides convincing evidence that markers of cachexia, including poor appetite [8,50], involuntary weight loss, in particular lean muscle [51,52], increased REE [53-55] and decreased albumin [55,56] are commonly observed in patients recovering from PFF.

Evidence exploring the inflammatory response post PFF surgery in this population group is scant. Previous research using fish oil as an intervention for cachexia has used the model of pancreatic cancer, however there have been considerable limitations in this research including heterogeneous samples representing mixed tumour types and severity, the use of variable and largely insufficient doses of EPA, and in some cases, the administration of fish oil for short durations [57]. Furthermore, the likelihood of demonstrating an effect with fish oil for patients recovering from PFF surgery is likely to be more favourable than patients with advanced cancer given the more acute nature of the injury. With evidence now suggesting a persistent inflammatory response in patients post PFF surgery, and that conventional nutrition support alone is unconvincing, in an attempt to dampen the inflammatory response, it is a logical extension to evaluate whether patients recovering from PFF surgery respond positively to fish oil as an intervention in concurrence with individualized nutrition support.

It is rare that PFF patients receive any significant level of nutrition support as part of standard care following their acute care admission. A major strength of the ATLANTIC trial is that it takes a comprehensive approach to nutrition management where all participants receive individual dietary counselling and nutrition education from an Accredited Practicing Dietitian. This includes the provision of dietary education and counselling in the way of dietary modifications, food fortification and oral supplementation to ensure individual energy, protein and other nutrient requirements are achieved. This is particularly critical given the lack of evidence that oral supplementation alone is effective at improving clinical outcomes in this patient group [16].

To minimize the devastating consequences of cachexia as well as secondary hospital admissions, it is critical that adequate rehabilitation and nutrition support services post PFF surgery are provided. The results of this trial will assist clinicians in the development of more effective and alternative treatment methods, such as an anti-inflammatory dose of fish oil post PFF surgery which may ultimately result in a reduction in systemic inflammation, weight loss, particularly muscle mass, improvements in nutritional status, mobility, independence and quality of life among these patients.

## Competing interests

The authors declare that they have no competing interests.

## Authors' contributions

MM, AY, LCo, RF, LCl, MJ, MC all contributed to the design of the study. MM co-ordinated and prepared drafts of the grant application and manuscript while AY and AV contributed to drafts of the manuscript and LCo, RF, LCl, MJ and MC all reviewed drafts for the grant application and manuscript. All authors have read and approve the publication of the final manuscript.

## Pre-publication history

The pre-publication history for this paper can be accessed here:

http://www.biomedcentral.com/1471-2318/10/76/prepub
